# Factors Influencing Virtual Reality as a Distraction Tool for Venipuncture in Children: Observational Pilot Feasibility Study

**DOI:** 10.2196/66656

**Published:** 2025-05-22

**Authors:** Chris Worth, Leyi Yang, Catherine Fullwood, Indraneel Banerjee

**Affiliations:** 1 Department of Paediatric Endocrinology, Royal Manchester Children's Hospital, Oxford Road, Manchester, M13 9WL, United Kingdom, 44 07837740913; 2 Research and Innovation, Manchester University NHS Foundation Trust, Manchester, United Kingdom; 3 Centre for Biostatistics, Division of Population Health, Health Services Research and Primary Care, School of Health Sciences, University of Manchester, Manchester, United Kingdom; 4 Faculty of Biology, Medicine and Health, University of Manchester, Manchester, United Kingdom

**Keywords:** virtual reality, pain, VR, computer-generated simulations, simulations, digital worlds, virtual environments, digital environments, virtual tour, venipuncture, children, VR headset, head-mounted display, cannulation, inpatients, questionnaires, pediatric care, phlebotomy

## Abstract

**Background:**

Virtual reality (VR) is increasingly used as a distraction tool for painful procedures in children. Studies have shown variable benefit but have not identified factors to maximize utility.

**Objective:**

This study aimed to undertake a feasibility study to investigate factors influencing virtual reality headset (VRH) utility for venipuncture and cannulation.

**Methods:**

Children admitted as inpatients were recruited and given a VRH during anticipated venipuncture or cannulation. Feedback from participants, parents, and operators was obtained through questionnaires and the Wong-Baker Faces Scale (WBFS).

**Results:**

Thirteen children of a target 32 (41%), aged a median of 7 (range 5-12) years, were recruited to the study; 9 (69%) parents and 7 (54%) participants reported a positive VR experience, found VRH comfortable, and wanted repeat application for future venisection or cannulation. However, patient recruitment was suboptimal at 41% (binomial 95% CI 24%‐59%) of the target, as busy operators undertaking venisection or cannulation on eligible patients were unable to spare time for VRH use during the COVID-19 pandemic. The preprocedure time spent with VRH was associated with participants’ positive experience of VR distraction (median 15, IQR 2.5‐50 vs 180, IQR 120-450) seconds (*P*=.02). Five (38.4%) participants removed the VRH prior to procedure; these were relatively young compared to those who continued VRH (median 6, IQR 5.00‐7.00 vs 10, IQR 6.75‐12.00 years), suggesting better acceptance of VR in older children. There was no significant difference in WBFS pain ratings before (median 0, IQR 0‐10) or after the procedure (median 0, IQR 0‐6), with many children choosing 0 (“no hurt”) at initial assessment. By contrast, parent and doctor or phlebotomist responses indicated that VR reduced pain and anxiety (n=9, 69%), in agreement with participant perception (n=7, 54%; Cohen κ=0.68).

**Conclusions:**

VR as a distraction tool in children is influenced by age and preprocedure familiarity, suggesting that the optimal use is in older children with greater cognitive and emotional maturity. Multidimensional feedback from participants, parents, and investigators should be obtained to test the true efficacy of VR in future studies.

## Introduction

### Overview

Virtual reality (VR) is a relatively new technology that is increasingly used as a distraction tool due to its immersive nature and capacity to engage multiple senses simultaneously [1]. VR utilizes the notion that attention is finite and can be diverted from pain and discomfort and therefore serves as effective cognitive distraction [2]. VR provides immersive engagement, isolating users from the real-world environment and associated pain and anxiety [1].

VR has been used as a nonpharmacological adjunct in pediatric care in a variety of clinical scenarios [3-9]. Children are often fearful of invasive procedures such as venisections and intravenous cannulation, which may need to be undertaken at frequent intervals depending on clinical needs; these procedures are necessary but reinforce and amplify pain and anxiety, adversely impacting on their hospital experience. Reduction of pain and anxiety is essential to continue effective clinical management for patient benefit and is currently provided by a range of pharmacological and nonpharmacological methods, including VR.

Studies have shown variable benefit of VR technology as a standalone tool, in comparison with alternative distraction techniques. However, most studies seeking to establish efficacy evaluated virtual reality headset (VRH) use in grouped study participants without qualification based on patient choice or other factors. A recent systematic review and meta-analysis [10] collated 11 randomized controlled trials in children under 12 years of age to conclude that VR was more useful than other nonpharmacological methods to relieve pain and anxiety for various procedures in children. Another recent meta-analysis [11] of 10 randomized controlled trials evaluated the efficacy of VR use in children aged 3 to 12 years to conclude favorably on the efficacy and benefit in pediatric venipuncture. However, results from such studies provide aggregated outcomes and are unable to investigate ancillary factors that encourage VRH adherence or dissuade participation. Therefore, such unqualified and indiscriminate evaluation may mask true benefits by failing to explore factors influencing the use of VR to maximize its distraction potential. There is a need to explore individual patient factors such as age, ethnicity, and prior experience of VR in additional studies that may qualify and add further strength to the adoption of this new technology in children’s health care.

Furthermore, the majority of previous VR studies have offered VRH to children during outpatient and planned procedures. The feasibility of VRH in the inpatient setting of a busy hospital, with less advance notice of procedures, has not been evaluated. It is important to investigate VRH use in high-pressure clinical areas to provide a more realistic and nuanced estimate of clinical benefit. We have undertaken a feasibility study to assess the practicalities of recruiting to such a study and to provide exploratory information around factors influencing VRH use in busy inpatient clinical areas in a children’s hospital.

### Aims

We aimed to explore the feasibility of VRH use and factors influencing VRH utility for venipuncture and cannulation within the inpatient pediatric setting.

## Methods

### Ethical Considerations

We undertook an observational, feasibility study (UK ethics approval 20/NE/0072 by the Health Research Authority; ClinicalTrials.gov NCT04472507) during the 2020‐2021 COVID-19 pandemic to review participant, parent and operator (doctor and phlebotomist) perspectives and investigate the optimal utility of VR distraction for inpatient venipuncture and cannulation. Study information was provided to ward-based operators undertaking venipuncture and cannulation. Potential patients and their parents also received study information prior to the procedure but the timing of the procedure was not confirmed, as it was dependent on clinical circumstances and operator availability. As patients were under 12 years old, parents (1 parent per child) consented for their participation in the study. Patients older than 6 years provided additional assent. Data were anonymized prior to write up. No compensation was provided to participants.

### Study Design and Analysis

Participants and parents (1 parent per child) were selected from all pediatric inpatients aged 5‐12 years, in a large tertiary hospital in the United Kingdom. All ethnic groups were eligible for participation. However, considering the unavailability of interpreters for various languages at the time of the COVID-19 pandemic, only English-speaking children and their families were selected. Operators performing inpatient venipuncture and cannulation informed the investigator team of eligible participants less than 6 hours before the procedure.

Prior experience of VRH was considered if a child had used a VRH in a nonclinical setting outside the hospital over the previous year. Information on prior experience was recorded by the investigator in a questionnaire administered to the parent. Experience of VRH use by a parent did not count as prior experience.

Participants were shown demonstrations of the OculusGo (Meta Reality Labs) VRH (and a choice of age-appropriate, single hand–operated software) and spent a median of 2 (range 0.1‐25.0) minutes using the VRH prior to their procedure, which lasted for 3 (range 0.1‐16.0) minutes. Each VRH was thoroughly cleaned after each procedure, which increased total procedural time. For the children’s feedback, the Wong-Baker Faces Scale (WBFS; scored from 0‐10) was chosen to record perceived pain before and after the procedure. Parent and operator feedback perspectives were captured by separate questionnaires and free text. The study design was principally quantitative and descriptive to provide the basis for the design of future larger trials using VR technologies. Therefore, no qualitative data were gathered in this feasibility study.

A target of 32 children to be recruited over a 4-week period was calculated to give 95% CI bounds of 56.6%‐88.5% (Clopper-Pearson exact) around the acceptability target of 75%. Statistical analysis was undertaken using IBM SPSS statistics, version 29.0 (descriptive data), and the statistical computing package R, version 4.2.1 (analytical data), without imputation for missing data. All data were presented with appropriate descriptive statistics: absolute values and percentages, means and SDs, or medians and IQRs or ranges. The Wilcox signed-rank test was used post hoc to assess differences in the nonparametric continuous variables—time and age—with subgroups defined by experience, while the demographic variables—gender and ethnicity—were assessed against experience using a Fisher exact test.

## Results

Thirteen children were recruited to the study during 3 week-long recruitment periods between January and May 2021. The median age at recruitment was 7 (range 5-12) years with a greater proportion of girls (n=8, 62%). Most participants (n=9, 69%) were White and of European ethnic origin, 2 were Black and of African origin, and 2 were ethnically South Asian (Table 1); all children and their families spoke English.

**Table 1.** Demographic information of all participants in the study.

		Value (n=13)
Age years, median (range)		7 (5-12)
Sex (female), n (%)		8 (62)
Ethnicity and race, n (%)		
	Asian Muslim	1 (8)
	Bangladeshi	1 (8)
	Black African	2 (15)
	White European	9 (69)
School stage, n (%)		
	Infant	4 (31)
	Junior	5 (38)
	Senior	4 (31)
Used virtual reality before, n (%)		6 (46)

Participant recruitment was challenging and considerably less than expected, with reluctance from busy operators who were concerned at introducing additional delay for eligible patients. Their feedback indicated the requirement for greater availability of VRH and advance planning. Recruitment peaked at 41% of the target (binomial 95% CI 24%‐59%), despite the addition of an extra week of recruitment beyond those initially planned.

Child-reported WBFS pain ratings did not demonstrate a change between preprocedure (median 0, IQR 0‐10) and postprocedure scores (median 0, IQR 0‐6), as many children chose 0 (“no hurt”) at the initial assessment. Nonetheless, more than half of participants (n=7, 54%) reported a positive experience, felt VRH was comfortable, and wanted to repeat the experience. Comments included “I loved it,” “it was really great” and “it was sick.” No child felt unwell during the procedure. There were no differences in participants’ VR distraction experience by either sex (*P*=.82) or ethnicity (*P*=.05). However, preprocedure time spent with VRH was found to be associated with participants’ positive experience of VR distraction (median 15, IQR 2.5‐50 vs 180, IQR 120-450 seconds; Wilcox signed-rank *P*=.02).

Although the VRH was provided for all participants, 5 (38%) children removed the VRH during the procedure citing that they “could not see mother” and “wanted to see arm” and that it “made them more anxious” and was “associated with blood sample.” Those who removed the VRH were relatively young compared to those who continued (median 6, IQR 5.00‐7.00 vs 10, IQR 6.75‐12.00 years). These children were all of primary school age, whereas those who engaged with the VRH were spread across both primary and secondary school age groups (UK school year for those who removed the VRH: median 2, IQR 1.00‐3.00 vs UK school year for those who remained engaged: median 6, IQR 3.75‐7.25; *P*=.03), suggesting better acceptability of VR in older children.

Over half of parents (n=8, 60%) perceived a reduction in both pain and anxiety in their children using VR distraction. This showed agreement with overall participant-reported positive experience and comfort and operator perception, with only 1 child reporting enjoyment contradicting operators’ perceptions. Parents reported a low perceived median pain score of 2 (range 0‐6), showing a moderate correlation (Cohen κ=0.57) with pre-VR participant-reported scores, although the same correlation was not seen postprocedure. Overall, parents felt their children had a positive VR experience during the procedure, in agreement with the participants’ perceptions (n=9, 69% and n=7, 54%, respectively; Cohen κ=0.68). Operators perceived VR to have reduced procedure time in only 3 (23%) children; however, they also noted that a majority of participants and parents had a positive experience (n=9, 69%).

## Discussion

### Principal Findings

Our exploratory results for the use of VR as a distraction tool for venipuncture and cannulation in children reported a positive experience in more than half of participants. While VRH use did not reduce procedure time, the overall experience of both children and their parents suggests benefits from use without adverse effects. While previous studies have reported aggregated benefits, our study has uncovered participant factors that have not been previously described, which are important for clinical application and for future study design. Importantly, our findings demonstrate the need for familiarization with the VRH and software prior to an invasive procedure. Our study also notes that more than a third of children removed their VRH; these children were relatively young and had greater parental attachment, suggesting that younger children and those with greater emotional needs may require additional strategies to remain connected in the VR environment to optimize VRH use. While children had individual reasons for removing their VRH, specific quotes from parent suggest reduced suitability in those with greater cognitive and emotional immaturity. By contrast, the acceptance and success of VRH in older children with greater psychological maturity is reassuring.

In previously published studies, VRH has been evaluated in all grouped participants [2,3,5,6] with suboptimal distraction utility, leading to the inevitable conclusion that further studies are required to achieve the full benefit [5]. Systematic reviews and meta-analyses have demonstrated aggregated benefit but have not identified individual factors that increase VR success [1,4]. Our study identified specific participant factors that suggest targeting VR for maximal gain: older age and prior experience and greater familiarity with a VRH. A multifaceted feedback approach, studying perspectives of parents, operators, and children, may assist the appreciation of different dimensions of pain, anxiety, and experience in VR distraction.

The study was designed as a feasibility study to assess the acceptability of the concept alongside a limited number of key variables in preparation for a future study in larger groups of children. Therefore, it has not been possible to assess the whole range of factors impacting VRH uptake and use. We expect to increase both the number of participants and the number of variables in a comprehensive study to critically test nuances around benefit and risk. Such a study will require an adequate sample size powered to detect differences among several variables, be standardized for VR software and content, and provide a preprocedure time for familiarization with VRH as well as consideration of additional operator time and how to minimize this in the inpatient setting.

We experienced reluctance from operators to engage with this study, citing the additional time required for VRH use. This extra time pressure must be considered when evaluating the feasibility of VRH in future inpatient pediatric settings. We also noted unusual responses from participants on the WBFS, with some children recording 0 (“no hurt”) at the initial assessment. While the use of the scale is justified in this study, we recognize its limitations, particularly in young children with learning disabilities. As the WBFS has a choice of only a few options, there may be limited sensitivity compared to other scales with a larger number of options. Additionally, children may have misinterpreted the crying faces on the WBFS to indicate emotional affect, instead of pain intensity [12,13].

Our study is limited by size owing to the reduced uptake of participants during the COVID-19 pandemic as well as the reluctance on behalf of operators undertaking venipuncture and cannulation services in a busy hospital ward setting. During the pandemic, patient-facing time was generally expedited to minimize potential spread of infection; this may have contributed to hesitance and reluctance of recruitment in both patients and operators. Therefore, conclusions drawn from this relatively small exploratory study will need to be carefully considered and tested for replication in larger cohorts alongside some preliminary qualitative data. Nonetheless, our data are important to assist the design of larger VR distraction trials to allow the redundancy of eligible participants being enrolled in the study and to optimize VRH use in high volume clinical settings.

The study is also constrained by the lack of a qualitative component that may have identified common themes from this relatively small sample. However, as the study was exploratory, we chose to focus on quantitative variables before designing a larger study with both quantitative and qualitative arms.

Despite study constraints, our results have uncovered real-world problems that will need to be addressed in future studies. Our study design did not provide a mandatory familiarization period, resulting in a rejection by some children, the majority of whom were under 7 years old. Therefore, for future studies, it would be important to stipulate a mandatory period to allow children and their parents to become familiar with the VRH and software, prior to study participation. Additionally, study design should consider strategies to ensure young children and those with greater parental attachment and emotional needs remain connected in the VR environment to optimize VRH use.

We acknowledge several sources of bias that may have influenced study findings. These include recruitment of relatively young patients, children with greater parental attachment, and those with or without previous experience of VRH use. It is possible that children and families with greater interest and experience of modern digital technology may have chosen to participate, thereby reducing the participation of those with a more cautious attitude to digital adoption. Although parental reports were mostly consistent with their children’s report, we recognize the possibility of reporting bias that may have influenced study findings. Another source of bias lies within the study population, the diversity of which was limited by COVID-19 restrictions on translators. We expect these biases to be addressed in the future design of more comprehensive studies.

We accept that VR technologies are expensive and not accessible to several families or even available in all hospitals. Some families may be inherently cautious of digital technologies and may resent the adoption of immersive distraction tools. VRH rejections in our study suggest that cultural and socioeconomic nuances may need to be addressed in future study designs and clinical application.

### Conclusion

Specific participant factors favor VR distraction in children. Future VR studies should target older children and those with greater cognitive and emotional maturity as the technology is more readily adopted in such groups. Furthermore, studies should introduce mandatory preprocedure VR familiarization time periods to ensure the standardization and optimal uptake of VRH. They should also include investigator and parent feedback in addition to child-provided pain ratings and questionnaires and ensure sufficient operational times are factored in the inpatient setting.

## Abbreviations

VR: virtual reality
VRH: virtual reality headset
WBFS: Wong-Baker Faces Scale
